# Supplementary material for: Multi-omics profiling of DNA methylation and gene expression alterations in human cocaine use disorder
Source: Transl Psychiatry. 2024 Oct 9;14:428. doi: 10.1038/s41398-024-03139-9 (PMC11464785; doi:10.1038/s41398-024-03139-9)
Supplement: Supplementary file 1 — Supplementary Figures [file 41398_2024_3139_MOESM1_ESM.pdf]

Supplementary Figures

Supplementary Figure S1 – Differential expression analysis in Brodmann Area 9

Supplementary Figure S2 – Weighted correlation network analysis reveals a cocaine-use disorder associated co-expression module in Brodmann Area 9

Supplementary Figure S3 – Protein-protein interaction network of module yellow hub genes

Supplementary Figure S4 – Variance explanation for DESeq2 model covariates across discovery and replication datasets

Supplementary Figure S5 – Drug repositioning analysis based on the differential gene expression signature in cocaine use disorder suggests glucocorticoid receptor targeting drugs as a potential pharmacotherapy

Supplementary Figure S6 – Integrated analysis of DNA methylation and gene expression data from Brodmann Area 9 using multi-omics factor analysis provides further evidence for synaptic signaling and metabolic alterations in cocaine use disorder

Supplementary Figure S7 – Integrative Gene Ontology enrichment analysis based on DNA methylation and expression signatures in Brodmann Area 9 reveals multiple functional modules associated with cocaine use disorder

Supplementary Figure S8 – Analysis workflow of the multi-omics analysis of DNA methylation (DNAm) and gene expression (RNA) in Brodmann Area 9 in cocaine use disorder

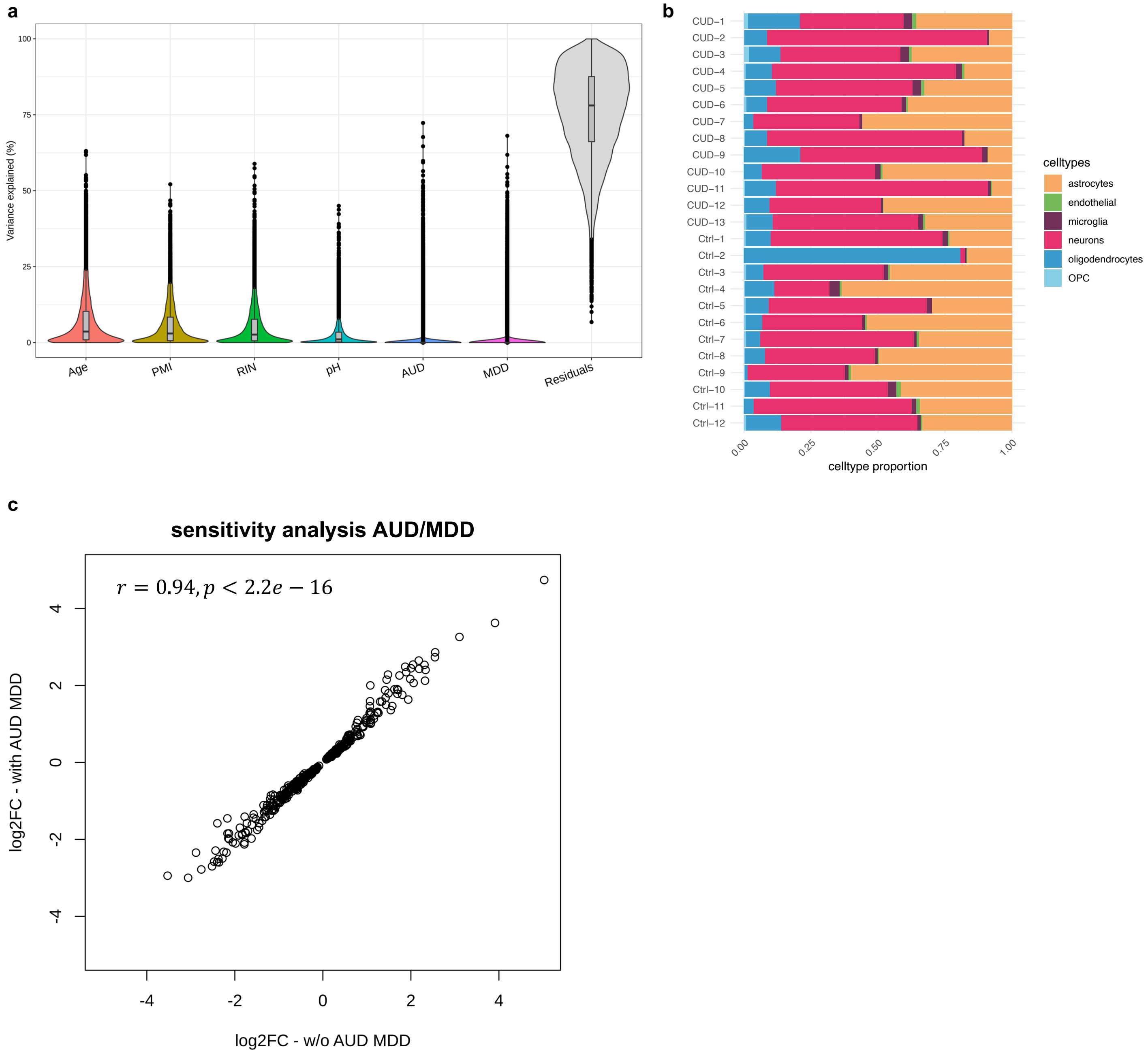

Supplementary Figure S1 – Differential expression analysis in Brodmann Area 9

a Variance partition analysis confirms the included covariates for the DE model in DESeq2 and reveals minimal explained variance for AUD and MDD. PMI=postmortem interval, RIN=RNA integrity number, pH=postmortem brain tissue pH value. **b** Cell type proportions in the N=25 postmortem human brain tissue samples from Brodmann Area 9 based on the estimation using CIBERSORT. CUD: N=13 cocaine use disorder samples, Ctrl: N=12 control samples. OPC=oligodendrocyte progenitor cell. **c** Correlation of log2FC estimates for nominal significant DE genes ($p<0.05$) in the sensitivity analysis that includes alcohol use disorder (AUD) and depressive disorder (MDD) as additional covariates into the DE model. A strong and highly significant Pearson correlation between log2FC estimates was observed ($r=0.94$, $p<2.2e-16$).

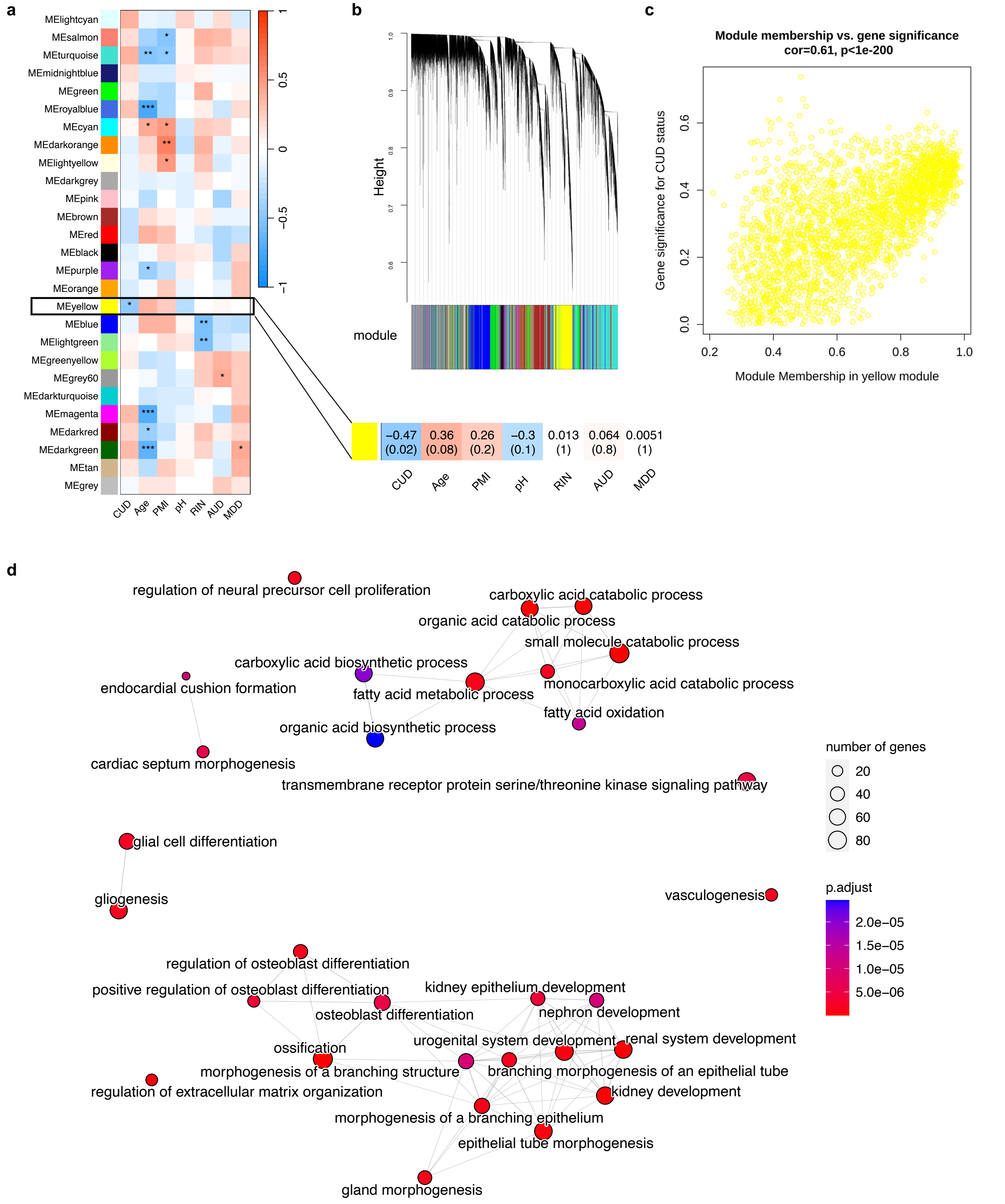

Supplementary Figure S2 – Weighted correlation network analysis reveals a cocaine-use disorder associated co-expression module in Brodmann Area 9

a Correlation of the N=27 identified co-expression modules from weighted correlation network analysis (WGCNA) with cocaine use disorder (CUD) status and other known phenotypes in the Brodmann Area 9 cohort. Pearson correlation of module eigengene with the phenotypic variable was color coded where red color indicates positive and blue color indicates negative correlation coefficients. Module yellow was significantly negatively correlated with CUD (r=-0.47, p=0.02). Significance of correlation is indicated using asterisks (*=p<0.05, **=p<0.01, ***=p<0.001). PMI=postmortem interval, pH=postmortem brain tissue pH value, RIN=RNA integrity number, AUD=alcohol use disorder status, MDD=depressive disorder status. **b** Dendrogram of genes and their assignment to co-expression modules in WGCNA. **c** Strong correlation of module membership and CUD gene significance for the N=2,517 genes in co-expression module yellow was identified (r=0.61, p<1e-200). **d** Results of the Gene Ontology (GO) enrichment analysis for the N=2,517 module yellow genes identified an overrepresentation in biological pathways related to development and fatty acid metabolism.

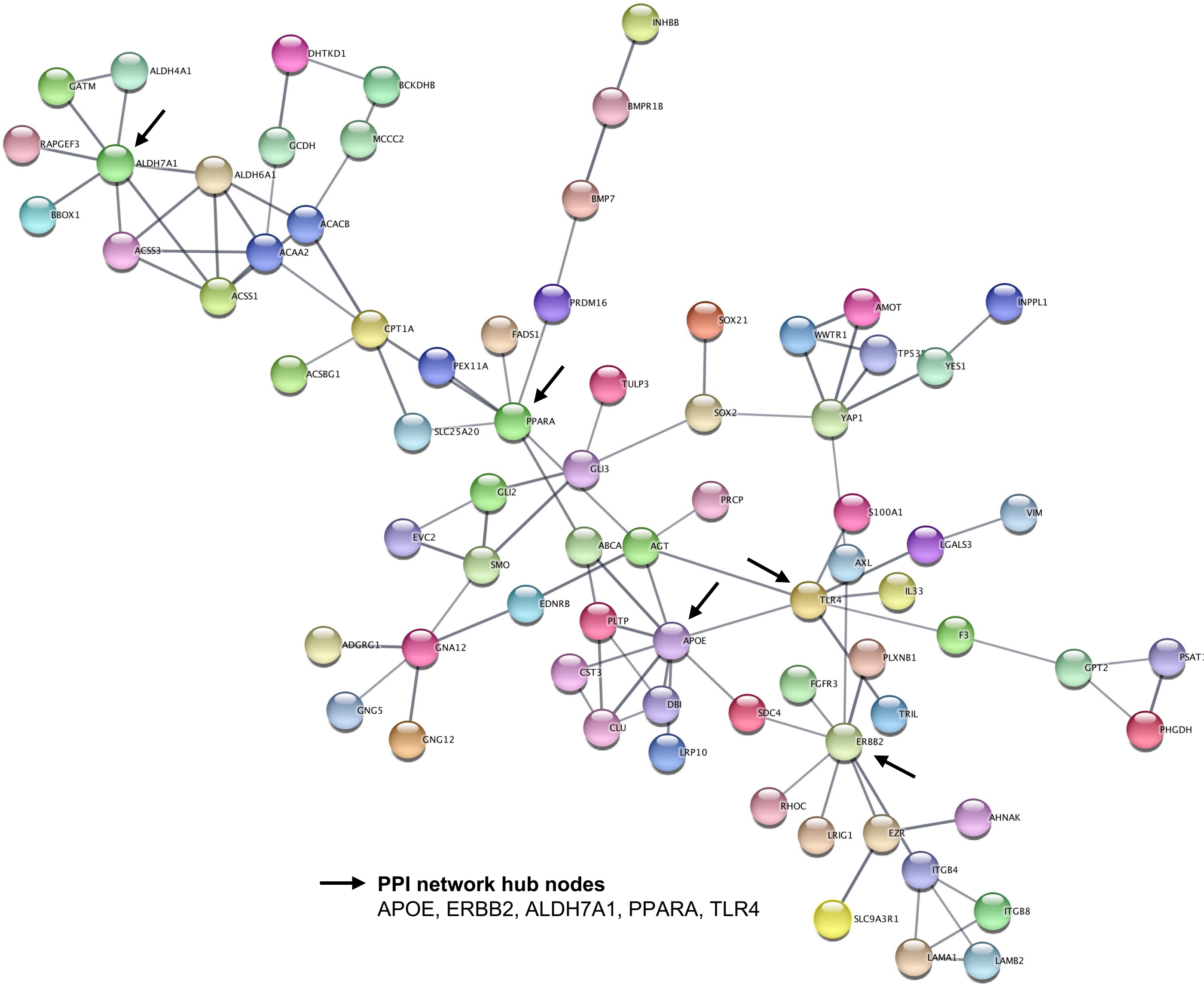

Supplementary Figure S3 – Protein-protein interaction network of module yellow hub genes

Protein-protein interaction STRING network of the top 10% module yellow hub genes reveals several highly connected network hub nodes such as APOE, ERBB2, and ALDH7A1, PPARA, and TLR4. Network hub nodes are highlighted by arrows.

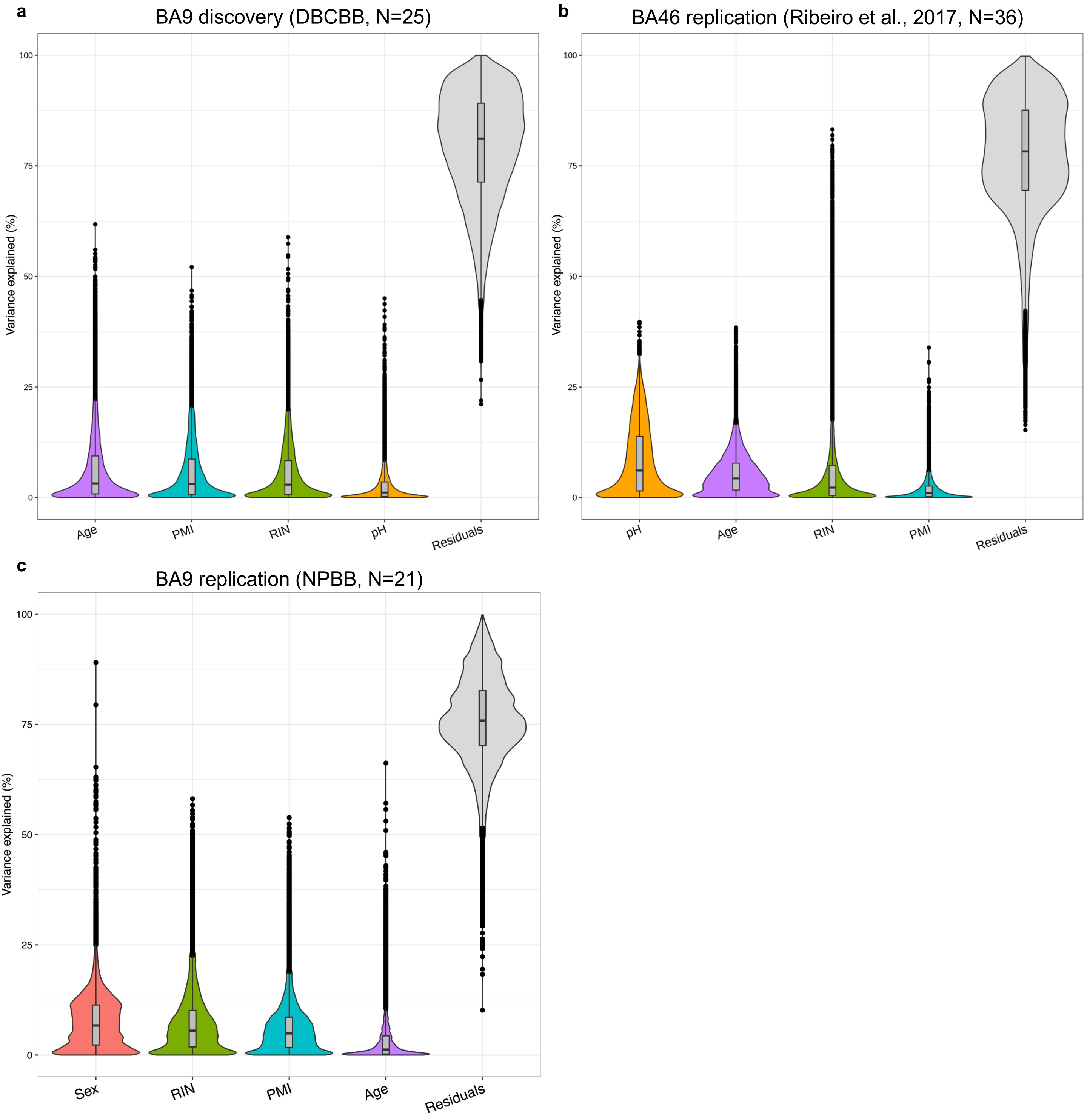

Supplementary Figure S4 – Variance explanation for DESeq2 model covariates across discovery and replication datasets
Variance partition analysis results for variables included as covariates into the DESeq2 differential gene expression analysis of **a** DBCBB RNA-seq discovery dataset from Brodmann Area 9 (BA9, N=25) **b** neuron-specific RNA-seq replication dataset from Brodmann Area 46 (N=36) and **c** BA9 RNA-seq replication dataset from NPBB (N=21).

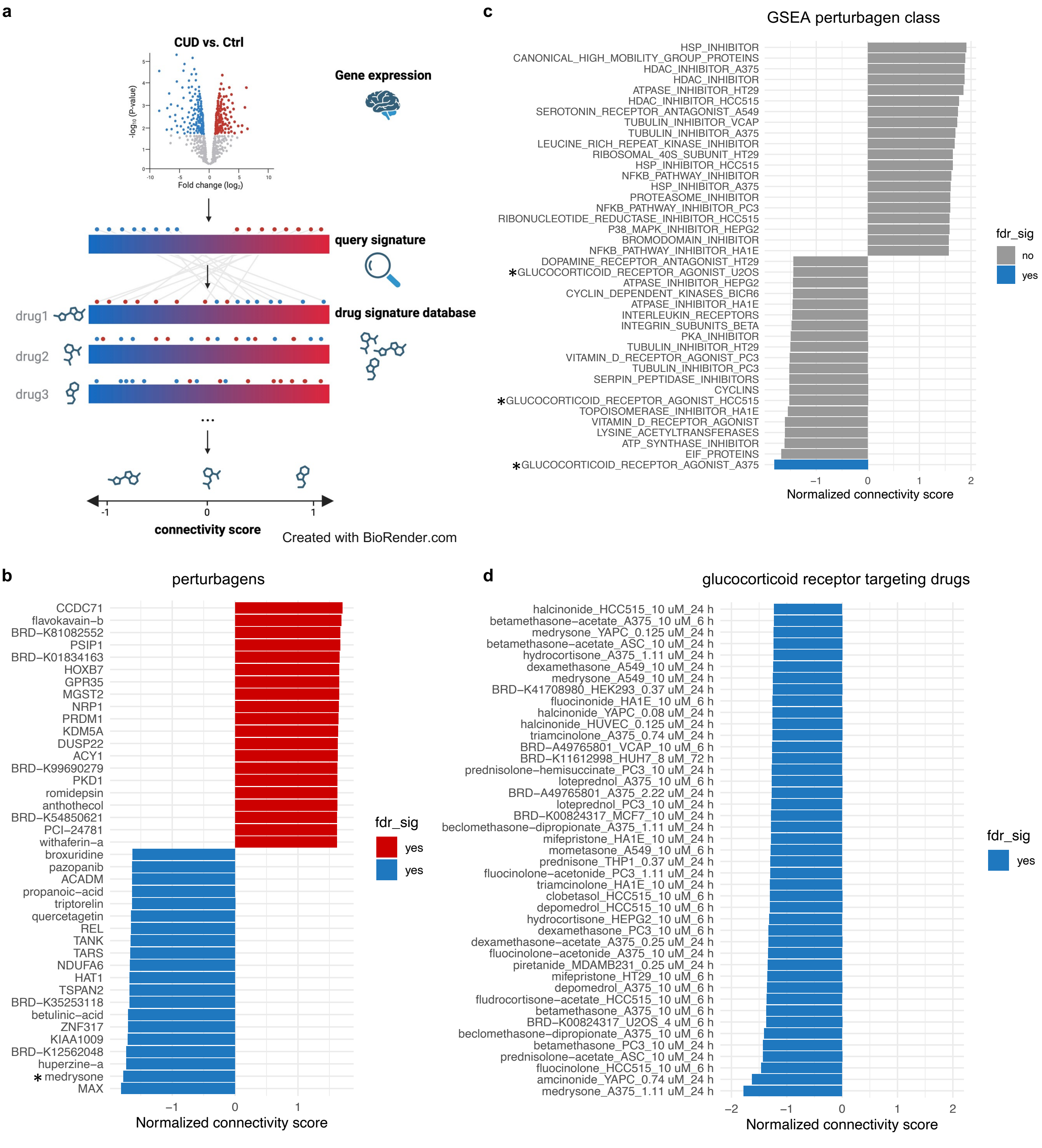

Supplementary Figure S5 – Drug repositioning analysis based on the differential gene expression signature in cocaine use disorder suggests glucocorticoid receptor targeting drugs as a potential pharmacotherapy
a Schematic overview of the drug repositioning analysis approach based on Connectivity Map (CMap) using the LINCS L1000 perturbagen reference database. The top N=150 upregulated and N=150 downregulated differentially expressed genes in cocaine use disorder were used as the query signature. **b** Results of the GSEA perturbagen class analysis from CMap. The top N=20 findings with positive and negative normalized connectivity score (NCS) are shown. Asterisks highlight findings related to glucocorticoid receptor agonists in different cell lines. **c** CMap results for individual perturbagen signatures that are most similar or dissimilar to the query signature. The top N=20 perturbagens with positive and negative NCS are shown. Asterisk highlights the glucocorticoid receptor targeting drug medrysone. **d** NCS for all glucocorticoid receptor targeting perturbagen drugs available in CMap were extracted indicating consistently significant negative NCS across cell lines compared to the query signature from Brodmann Area 9. Blue color indicates significance (fdr_sig, q<0.05) after FDR-correction for multiple testing.

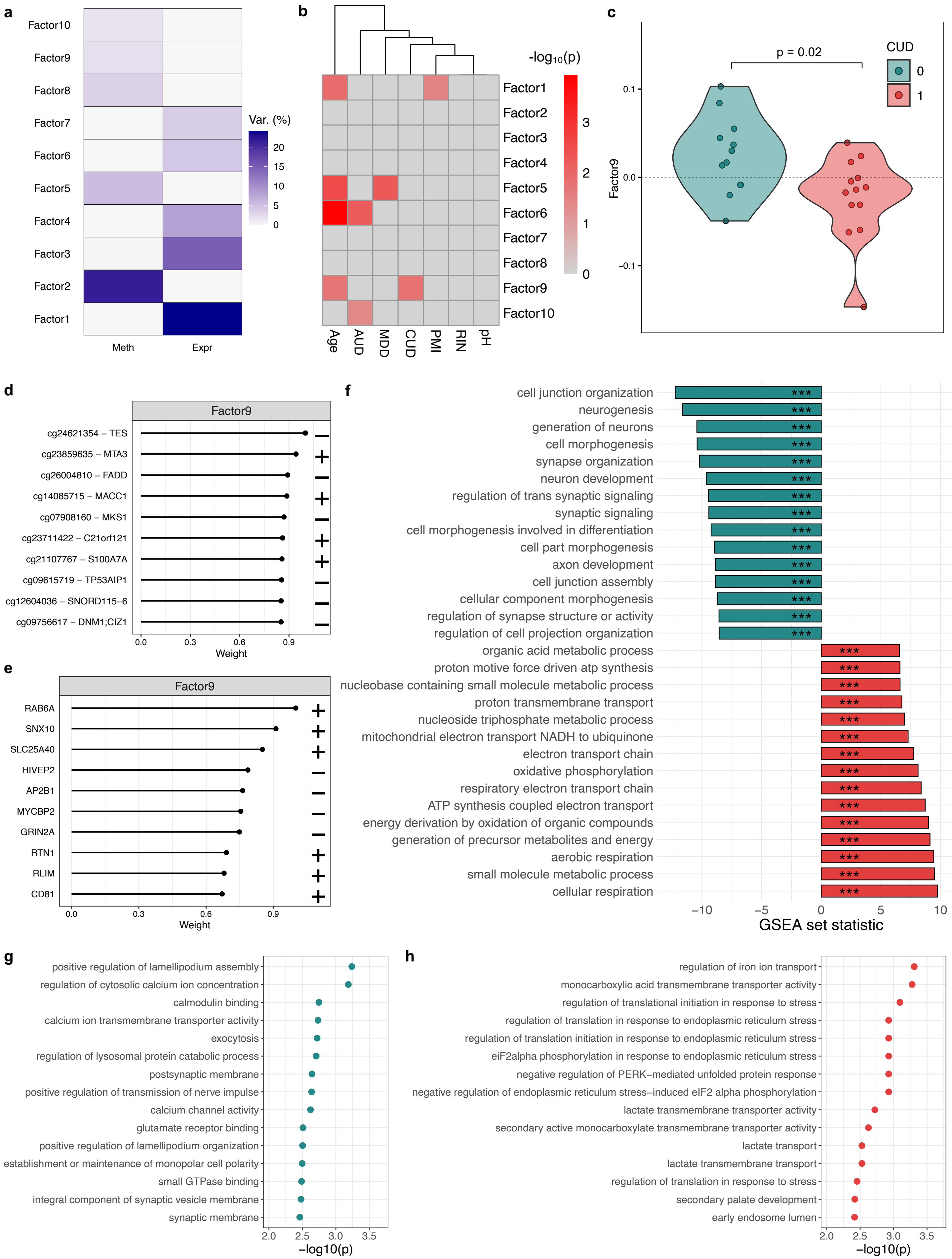

a
Factor10
Factor9
Factor8
Factor7
Factor6
Factor5
Factor4
Factor3
Factor2
Factor1
Meth
Expr
Var. (%)
20
15
10
5
0
b
-log10(p)
3
2
1
0
Factor1
Factor2
Factor3
Factor4
Factor5
Factor6
Factor7
Factor8
Factor9
Factor10
Age
AUD
MDD
CUD
PMI
RIN
pH
c
p = 0.02
CUD
0
1
Factor9
0.1
0.0
−0.1
d
Factor9
cg24621354 – TES
cg23859635 – MTA3
cg26004810 – FADD
cg14085715 – MACC1
cg07908160 – MKS1
cg23711422 – C21orf121
cg21107767 – S100A7A
cg09615719 – TP53AIP1
cg12604036 – SNORD115–6
cg09756617 – DNM1;CIZ1
0.0
0.3
0.6
0.9
Weight
e
Factor9
RAB6A
SNX10
SLC25A40
HIVEP2
AP2B1
MYCBP2
GRIN2A
RTN1
RLIM
CD81
0.0
0.3
0.6
0.9
Weight
f
cell junction organization
neurogenesis
generation of neurons
cell morphogenesis
synapse organization
neuron development
regulation of trans synaptic signaling
synaptic signaling
cell morphogenesis involved in differentiation
cell part morphogenesis
axon development
cell junction assembly
cellular component morphogenesis
regulation of synapse structure or activity
regulation of cell projection organization
organic acid metabolic process
proton motive force driven atp synthesis
nucleobase containing small molecule metabolic process
proton transmembrane transport
nucleoside triphosphate metabolic process
mitochondrial electron transport NADH to ubiquinone
electron transport chain
oxidative phosphorylation
respiratory electron transport chain
ATP synthesis coupled electron transport
energy derivation by oxidation of organic compounds
generation of precursor metabolites and energy
aerobic respiration
small molecule metabolic process
cellular respiration
***
−10
−5
0
5
10
GSEA set statistic
g
positive regulation of lamellipodium assembly
regulation of cytosolic calcium ion concentration
calmodulin binding
calcium ion transmembrane transporter activity
exocytosis
regulation of lysosomal protein catabolic process
postsynaptic membrane
positive regulation of transmission of nerve impulse
calcium channel activity
glutamate receptor binding
positive regulation of lamellipodium organization
establishment or maintenance of monopolar cell polarity
small GTPase binding
integral component of synaptic vesicle membrane
synaptic membrane
2.0
2.5
3.0
3.5
−log10(p)
h
regulation of iron ion transport
monocarboxylic acid transmembrane transporter activity
regulation of translational initiation in response to stress
regulation of translation in response to endoplasmic reticulum stress
regulation of translation initiation in response to endoplasmic reticulum stress
eiF2alpha phosphorylation in response to endoplasmic reticulum stress
negative regulation of PERK–mediated unfolded protein response
negative regulation of endoplasmic reticulum stress–induced eIF2 alpha phosphorylation
lactate transmembrane transporter activity
secondary active monocarboxylate transmembrane transporter activity
lactate transport
lactate transmembrane transport
regulation of translation in response to stress
secondary palate development
early endosome lumen
2.0
2.5
3.0
3.5
−log10(p)

Supplementary Figure S6 – Integrated analysis of DNA methylation and gene expression data from Brodmann Area 9 using multi-omics factor analysis provides further evidence for synaptic signaling and metabolic alterations in cocaine use disorder

a Variance explanation (in %) in the DNA methylation (Meth, N=20,000 most variable promoter CpG sites) and expression dataset (Expr, N=20,000 most variable genes) for each of the identified factors from multi-omics factor analysis (MOFA). **b** Association of MOFA factors with cocaine use disorder (CUD) and known covariates in the postmortem human brain cohort. AUD = alcohol use disorder, MDD/NOS = major depressive disorder/depressive disorder not otherwise specified, PMI= postmortem interval, RIN = RNA integrity number, pH = brain tissue pH value. Red color indicates statistically significant association with the phenotype ($p<0.05$). **c** Significant difference in factor 9 values was observed between CUD cases and Ctrl ($p=0.02$). Top 10 **d** CpG sites (Meth) and **e** genes (Expr) having the strongest absolute weights on factor 9. +: positive weight, -: negative weight. **f** Results of the gene set enrichment analysis (GSEA) on the expression weights on factor 9 separated by positive and negative weights. Significance estimates inside bars are FDR-adjusted q-values (***, $q<0.001$). For the DNA methylation dataset, results of the GO enrichment analysis for CpG sites with strongest **g** negative weights and **h** positive weights on factor 9 are shown.

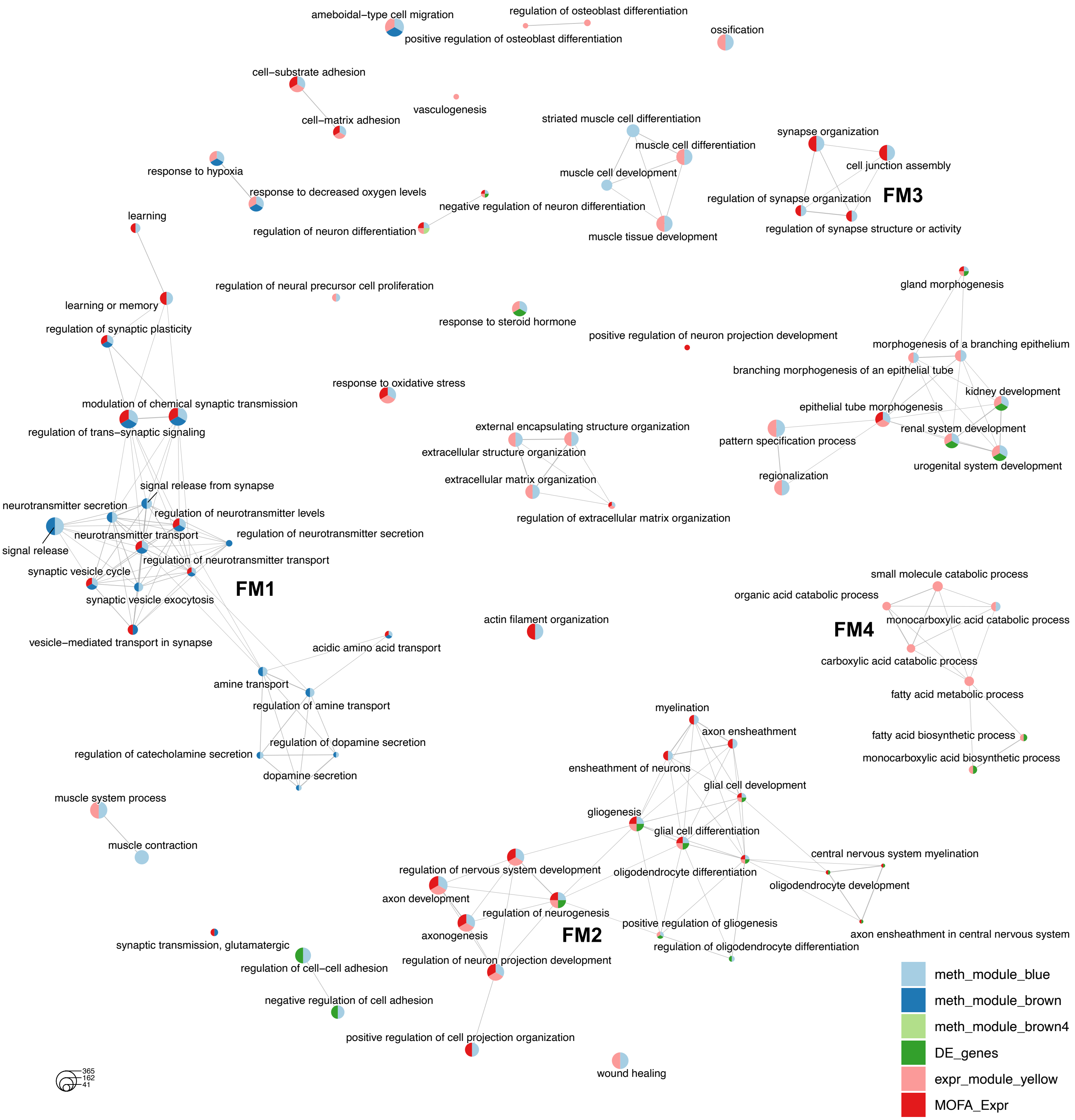

Supplementary Figure S7 – Integrative Gene Ontology enrichment analysis based on DNA methylation and expression signatures in Brodmann Area 9 reveals multiple functional modules associated with cocaine use disorder

Enrichment map of the Gene Ontology (GO) enrichment analysis using cocaine use disorder (CUD) associated gene lists derived from individual and integrative analyses of DNA methylation and gene expression signatures in Brodmann Area 9. Convergent evidence at the biological pathway level leads to the emergence of functional modules (FM) with interconnected GO terms where statistically significant (q<0.05) enrichment was detected for multiple gene lists.

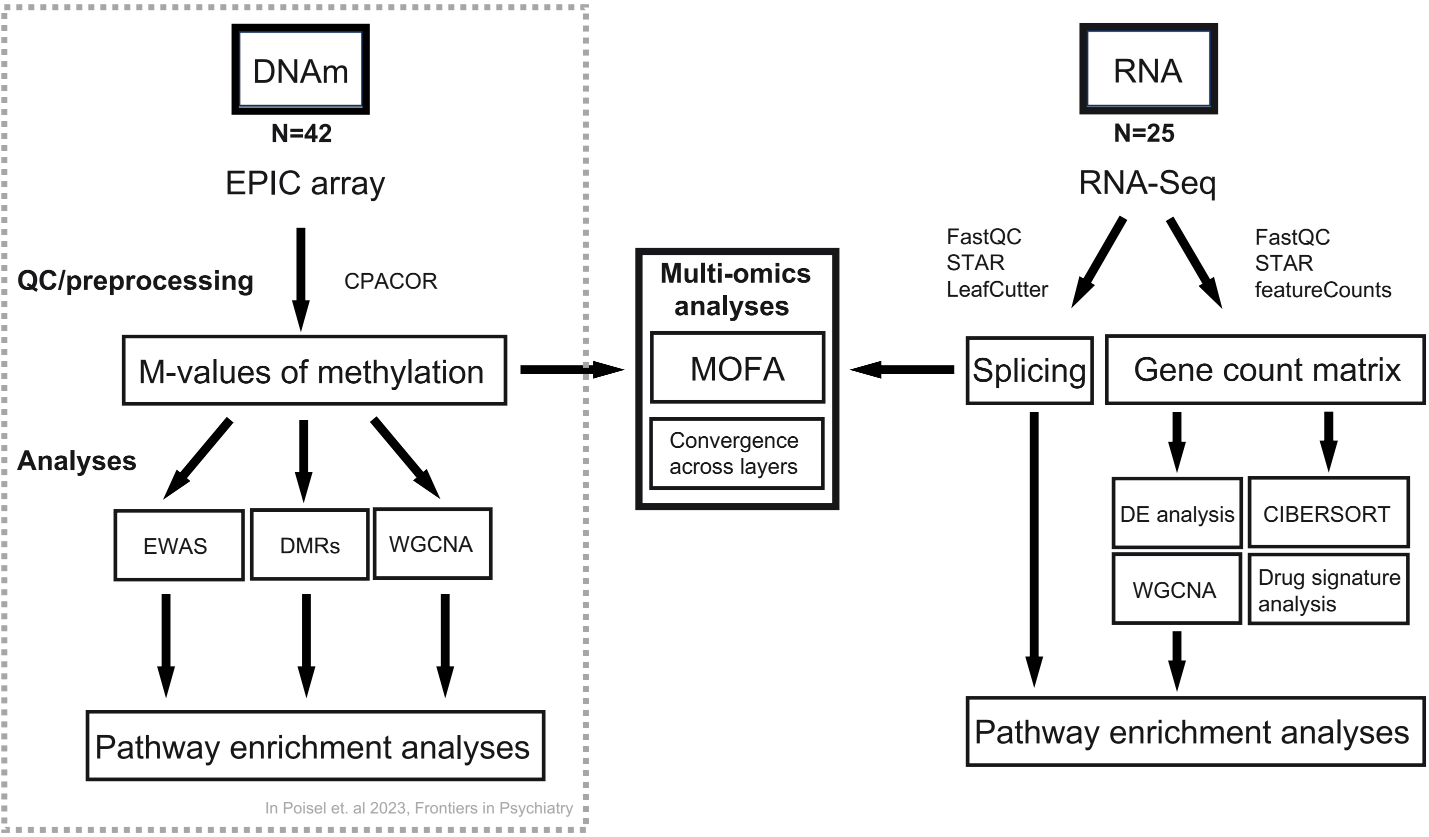

Supplementary Figure S8 – Analysis workflow of the multi-omics analysis of DNA methylation (DNAm) and gene expression (RNA) in Brodmann Area 9 in cocaine use disorder

Analysis of the DNA methylation (DNAm) dataset from the N=42 Brodmann Area 9 samples has been performed in Poisel et al., 2023. Transcriptome-wide gene expression analysis from RNA-sequencing in a subset of N=25 individuals of this cohort and multi-omics data integration is performed in the present study. CPACOR=Control Probe Adjustment and reduction of global CORrelation (Lehne et al., 2015), EWAS=epigenome-wide association study, DMRs=differentially methylated regions, WGCNA=weighted gene co-expression network analysis, MOFA=multi omics factor analysis, DE=differential expression. The DNAm analysis was previously published in Poisel et al., Frontiers in Psychiatry, 2023.
